# (2*E*)-1-(3-Bromo­phen­yl)-3-(6-meth­oxy-2-naphth­yl)prop-2-en-1-one

**DOI:** 10.1107/S1600536810035117

**Published:** 2010-09-11

**Authors:** William T. A. Harrison, A. N. Mayekar, H. S. Yathirajan, B. Narayana

**Affiliations:** a Department of Chemistry, University of Aberdeen, Meston Walk, Aberdeen AB24 3UE, Scotland; b Department of Studies in Chemistry, University of Mysore, Manasagangotri, Mysore 570 006, India; c SeQuent Scientific Limited, New Mangalore 575 011, India; d Department of Chemistry, Mangalore University, Mangalagangotri 574 199, India

## Abstract

In the title compound, C_20_H_15_BrO_2_, the prop-2-en-1-one fragment is substanti­ally twisted [C—C—C—O = 23.0 (11)°]. The dihedral angle between the benzene and naphthalene rings is 44.28 (13)°. The only possible directional inter­actions in the crystal are weak C—H⋯π contacts, which generate (001) sheets.

## Related literature

For related structures, see: Yathirajan *et al.* (2007*a* ▶,*b* ▶); Jasinski *et al.* (2009 ▶). For background to the non-linear optical properties of chalcones, see: Sarojini *et al.* (2006 ▶). For reference structural data, see: Allen *et al.* (1987 ▶).
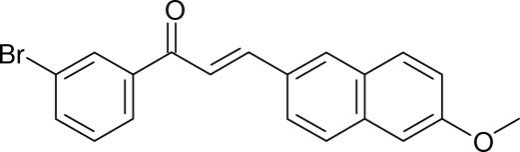


## Experimental

### 

#### Crystal data

C_20_H_15_BrO_2_
*M* *_r_* = 367.23
Orthorhombic, 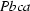

*a* = 14.0955 (14) Å
*b* = 6.1295 (6) Å
*c* = 36.119 (4) Å
*V* = 3120.6 (5) Å^3^
*Z* = 8
Mo *K*α radiation
μ = 2.64 mm^−1^
*T* = 120 K
0.11 × 0.09 × 0.03 mm

#### Data collection

Nonius KappaCCD diffractometer
Absorption correction: multi-scan (*SADABS*; Bruker, 2003 ▶) *T*_min_ = 0.760, *T*_max_ = 0.925
28579 measured reflections
3545 independent reflections
1719 reflections with *I* > 2σ(*I*)
*R*_int_ = 0.228

#### Refinement

*R*[*F* ^2^ > 2σ(*F* ^2^)] = 0.082
*wR*(*F* ^2^) = 0.163
*S* = 1.05
3545 reflections
209 parameters
H-atom parameters constrained
Δρ_max_ = 0.61 e Å^−3^
Δρ_min_ = −0.63 e Å^−3^

### 

Data collection: *COLLECT* (Nonius, 1998 ▶); cell refinement: *SCALEPACK* (Otwinowski & Minor, 1997 ▶); data reduction: *DENZO* (Otwinowski & Minor, 1997 ▶), *SCALEPACK* and *SORTAV* (Blessing, 1995 ▶); program(s) used to solve structure: *SHELXS97* (Sheldrick, 2008 ▶); program(s) used to refine structure: *SHELXL97* (Sheldrick, 2008 ▶); molecular graphics: *ORTEP-3* (Farrugia, 1997 ▶); software used to prepare material for publication: *SHELXL97*.

## Supplementary Material

Crystal structure: contains datablocks I, global. DOI: 10.1107/S1600536810035117/tk2699sup1.cif

Structure factors: contains datablocks I. DOI: 10.1107/S1600536810035117/tk2699Isup2.hkl

Additional supplementary materials:  crystallographic information; 3D view; checkCIF report

## Figures and Tables

**Table 1:** Hydrogen-bond geometry (Å, °) *Cg*2 is the centroid of the C3–C8 ring.

*D*—H⋯*A*	*D*—H	H⋯*A*	*D*⋯*A*	*D*—H⋯*A*
C4—H4⋯*Cg*2^i^	0.95	2.70	3.432 (6)	134
C7—H7⋯*Cg*2^ii^	0.95	2.80	3.520 (6)	134

Symmetry codes: (i) 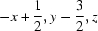; (ii) 
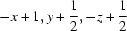
.

**Fig. 1.**
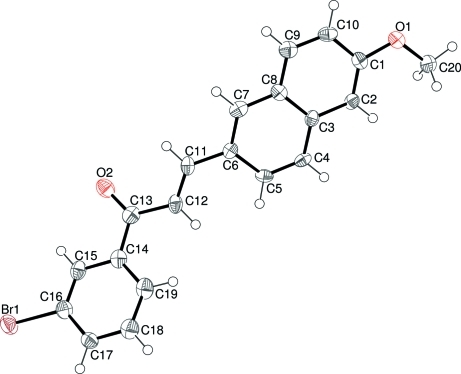
View of the molecular structure of (I) showing 50% displacement ellipsoids (arbitrary spheres for the H atoms).

**Fig. 2.**
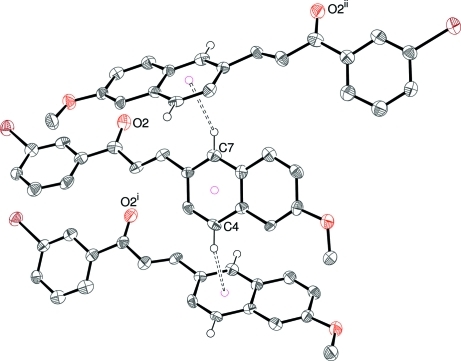
Partial packing diagram for (I) showing the possible weak C—H···π contacts. All H atoms except H4 and H7 omitted for clarity. Symmetry codes: (i) 1/2–x, y–1/2, z; (ii) 1–x, 1/2 + y, 1/2–z.

## supplementary crystallographic information

### Comment

The title compound, (I), (Fig. 1), was prepared as part of our ongoing studies (Yathirajan *et al.*, 2007*a*,*b*; Jasinski *et al.*, 2009) of substituted phenyl/naphthyl chalcone derivatives as possible candidates for non-linear optical materials (Sarojini *et al.*, 2006). However, (I) crystallizes in a centrosymmetric space group, thus its second-harmonic generation (SHG) response must be zero.

The prop-2-en-1-one (enone) fragment in (I) is substantially twisted, as indicated by the C11—C12—C13—O2 torsion angle of 23.0 (11)°. The dihedral angle between the aromatic ring systems is 44.28 (13)°. Equivalent data for related structures are as follows: (2*E*)-1-(2,4-dichlorophenyl)-3-(6-methoxy-2-naphthyl)prop-2-en-1-one (Yathirajan *et al.*, 2007*a*): -10.9 (2) and 44.94 (4)°; (2*E*)-3-(6-methoxy-2-naphthyl)-1-phenylprop-2-en-1-one (Yathirajan *et al.*, 2007*b*): -15.9 (4) and 14.9 (8)°; (2*E*)-1-(2-hydroxyphenyl)-3-(6-methoxy-2-naphthyl)prop-2-en-1-one (Jasinski *et al.*, 2009): -14.9 (2) and 31.7 (3)°. Otherwise, the bond lengths for (I) fall within their expected ranges (Allen *et al.*, 1987).

In the crystal of (I), the only possible directional interactions between molecules are weak C—H···π contacts in which the C3–C8 ring of the naphthyl moiety provides both the C—H donor groups and the aromatic acceptor surface (Table 1, Fig. 2). Together, these generate (001) sheets.

### Experimental

To a thoroughly stirred solution of 6-methoxy-2-naphthaldehyde (1.86 g, 0.01 mol) and 3-bromoacetophenone (1.99 g, 0.01 mol) in 25 ml methanol, 5 ml of 40% KOH solution was added. The reaction mixture was stirred overnight and the solid separated was collected by filtration. The product obtained was recrystallized from methanol. Colourless slabs of (I) were grown by the slow evaporation of the ethylacetate solution (m.p. 427–429 K).

### Refinement

The crystal studied was a weak scatterer, which may correlate with the high *R*_int_ value. The hydrogen atoms were geometrically placed (C—H = 0.95–0.98 Å) and refined as riding with *U*_iso_(H) = 1.2*U*_eq_(C) or 1.5*U*_eq_(methyl C). A rotating rigid-group model was applied to the methyl group.

### Crystal data

**Table:**

C_20_H_15_BrO_2_	*F*(000) = 1488
*M**_r_* = 367.23	*D*_x_ = 1.563 Mg m^−^^3^
Orthorhombic, *P**b**c**a*	Mo *K*α radiation, λ = 0.71073 Å
Hall symbol: -P 2ac 2ab	Cell parameters from 55128 reflections
*a* = 14.0955 (14) Å	θ = 2.9–27.5°
*b* = 6.1295 (6) Å	µ = 2.64 mm^−^^1^
*c* = 36.119 (4) Å	*T* = 120 K
*V* = 3120.6 (5) Å^3^	Slab, colourless
*Z* = 8	0.11 × 0.09 × 0.03 mm

### Data collection

**Table:**

Nonius KappaCCD diffractometer	3545 independent reflections
Radiation source: fine-focus sealed tube	1719 reflections with *I* > 2σ(*I*)
graphite	*R*_int_ = 0.228
ω and φ scans	θ_max_ = 27.5°, θ_min_ = 3.1°
Absorption correction: multi-scan (*SADABS*; Bruker, 2003)	*h* = −18→18
*T*_min_ = 0.760, *T*_max_ = 0.925	*k* = −7→7
28579 measured reflections	*l* = −46→46

### Refinement

**Table:**

Refinement on *F*^2^	Primary atom site location: structure-invariant direct methods
Least-squares matrix: full	Secondary atom site location: difference Fourier map
*R*[*F*^2^ > 2σ(*F*^2^)] = 0.082	Hydrogen site location: inferred from neighbouring sites
*wR*(*F*^2^) = 0.163	H-atom parameters constrained
*S* = 1.05	*w* = 1/[σ^2^(*F*_o_^2^) + (0.0343*P*)^2^ + 12.2862*P*] where *P* = (*F*_o_^2^ + 2*F*_c_^2^)/3
3545 reflections	(Δ/σ)_max_ < 0.001
209 parameters	Δρ_max_ = 0.61 e Å^−^^3^
0 restraints	Δρ_min_ = −0.63 e Å^−^^3^

### Special details

**Table:**

Geometry. All e.s.d.'s (except the e.s.d. in the dihedral angle between two l.s. planes) are estimated using the full covariance matrix. The cell e.s.d.'s are taken into account individually in the estimation of e.s.d.'s in distances, angles and torsion angles; correlations between e.s.d.'s in cell parameters are only used when they are defined by crystal symmetry. An approximate (isotropic) treatment of cell e.s.d.'s is used for estimating e.s.d.'s involving l.s. planes.
Refinement. Refinement of *F*^2^ against ALL reflections. The weighted *R*-factor *wR* and goodness of fit *S* are based on *F*^2^, conventional *R*-factors *R* are based on *F*, with *F* set to zero for negative *F*^2^. The threshold expression of *F*^2^ > σ(*F*^2^) is used only for calculating *R*-factors(gt) *etc*. and is not relevant to the choice of reflections for refinement. *R*-factors based on *F*^2^ are statistically about twice as large as those based on *F*, and *R*- factors based on ALL data will be even larger.

### Fractional atomic coordinates and isotropic or equivalent isotropic displacement parameters (Å^2^)

**Table:**

	*x*	*y*	*z*	*U*_iso_*/*U*_eq_	
C1	0.3881 (4)	0.3963 (12)	0.14070 (18)	0.0269 (16)	
C2	0.3536 (5)	0.2944 (11)	0.17160 (18)	0.0260 (16)	
H2	0.3224	0.1576	0.1695	0.031*	
C3	0.3647 (4)	0.3940 (11)	0.20683 (18)	0.0244 (15)	
C4	0.3332 (4)	0.2939 (11)	0.23966 (18)	0.0247 (16)	
H4	0.3057	0.1526	0.2384	0.030*	
C5	0.3411 (4)	0.3933 (11)	0.27329 (17)	0.0257 (15)	
H5	0.3192	0.3202	0.2949	0.031*	
C6	0.3820 (4)	0.6074 (12)	0.27654 (18)	0.0226 (15)	
C7	0.4144 (4)	0.7064 (11)	0.24441 (19)	0.0266 (17)	
H7	0.4418	0.8477	0.2460	0.032*	
C8	0.4083 (4)	0.6051 (12)	0.20954 (18)	0.0249 (16)	
C9	0.4425 (5)	0.7045 (12)	0.1768 (2)	0.0316 (18)	
H9	0.4724	0.8432	0.1782	0.038*	
C10	0.4332 (4)	0.6034 (12)	0.14329 (19)	0.0290 (16)	
H10	0.4569	0.6718	0.1216	0.035*	
C11	0.3820 (4)	0.7262 (12)	0.31156 (18)	0.0262 (18)	
H11	0.4035	0.8730	0.3107	0.031*	
C12	0.3550 (5)	0.6518 (11)	0.34459 (18)	0.0290 (17)	
H12	0.3356	0.5039	0.3468	0.035*	
C13	0.3544 (5)	0.7926 (12)	0.3780 (2)	0.0305 (17)	
C14	0.3642 (4)	0.6878 (12)	0.4150 (2)	0.0276 (17)	
C15	0.3486 (5)	0.8158 (12)	0.44671 (19)	0.0312 (17)	
H15	0.3274	0.9622	0.4442	0.037*	
C16	0.3638 (5)	0.7308 (12)	0.4810 (2)	0.0313 (17)	
C17	0.3927 (5)	0.5156 (12)	0.48559 (19)	0.0290 (18)	
H17	0.4014	0.4555	0.5096	0.035*	
C18	0.4085 (5)	0.3918 (13)	0.4543 (2)	0.0363 (18)	
H18	0.4305	0.2461	0.4571	0.044*	
C19	0.3934 (5)	0.4728 (12)	0.4191 (2)	0.035 (2)	
H19	0.4029	0.3827	0.3981	0.042*	
C20	0.3351 (5)	0.1184 (12)	0.10028 (19)	0.0400 (19)	
H20A	0.3318	0.0822	0.0739	0.060*	
H20B	0.2707	0.1298	0.1103	0.060*	
H20C	0.3697	0.0036	0.1135	0.060*	
O1	0.3827 (3)	0.3202 (8)	0.10487 (13)	0.0351 (13)	
O2	0.3495 (4)	0.9934 (9)	0.37483 (13)	0.0361 (12)	
Br1	0.34948 (6)	0.90763 (13)	0.52413 (2)	0.0403 (3)	

### Atomic displacement parameters (Å^2^)

**Table:**

	*U*^11^	*U*^22^	*U*^33^	*U*^12^	*U*^13^	*U*^23^
C1	0.024 (4)	0.033 (4)	0.024 (4)	0.007 (4)	−0.003 (3)	−0.002 (4)
C2	0.027 (4)	0.023 (4)	0.027 (4)	−0.005 (4)	−0.011 (4)	−0.001 (3)
C3	0.022 (4)	0.022 (4)	0.029 (4)	0.002 (4)	0.000 (3)	−0.003 (4)
C4	0.021 (4)	0.019 (4)	0.034 (4)	0.002 (3)	0.004 (3)	0.006 (3)
C5	0.022 (3)	0.031 (4)	0.024 (4)	−0.002 (4)	0.003 (3)	0.008 (4)
C6	0.014 (3)	0.023 (4)	0.030 (4)	−0.001 (3)	−0.002 (3)	−0.001 (4)
C7	0.013 (3)	0.029 (4)	0.038 (5)	−0.001 (3)	0.002 (3)	0.004 (4)
C8	0.018 (3)	0.031 (4)	0.026 (4)	0.005 (4)	−0.001 (3)	0.000 (4)
C9	0.027 (4)	0.034 (4)	0.034 (5)	0.002 (4)	0.003 (4)	0.003 (4)
C10	0.022 (4)	0.032 (4)	0.033 (4)	0.000 (4)	0.003 (3)	0.009 (4)
C11	0.019 (4)	0.028 (4)	0.031 (4)	0.001 (3)	0.000 (3)	−0.009 (4)
C12	0.024 (4)	0.031 (4)	0.032 (4)	−0.006 (4)	0.000 (4)	−0.009 (3)
C13	0.021 (4)	0.032 (5)	0.038 (4)	−0.001 (4)	0.011 (4)	−0.001 (4)
C14	0.016 (4)	0.033 (4)	0.034 (4)	−0.002 (3)	0.002 (3)	−0.004 (4)
C15	0.025 (4)	0.030 (4)	0.039 (4)	−0.004 (4)	0.002 (4)	−0.007 (4)
C16	0.028 (4)	0.034 (4)	0.032 (4)	0.003 (3)	0.003 (4)	−0.004 (4)
C17	0.027 (4)	0.034 (4)	0.026 (4)	−0.003 (3)	−0.001 (3)	0.002 (3)
C18	0.030 (4)	0.038 (5)	0.041 (5)	0.000 (4)	−0.005 (4)	−0.003 (5)
C19	0.023 (4)	0.040 (5)	0.042 (5)	−0.001 (3)	−0.011 (4)	−0.007 (4)
C20	0.059 (5)	0.033 (4)	0.029 (4)	−0.007 (4)	−0.005 (4)	−0.004 (4)
O1	0.045 (3)	0.035 (3)	0.026 (3)	−0.002 (2)	0.001 (2)	−0.001 (3)
O2	0.034 (3)	0.044 (3)	0.031 (3)	0.006 (3)	0.001 (3)	−0.002 (3)
Br1	0.0471 (4)	0.0440 (5)	0.0298 (4)	0.0048 (5)	0.0016 (4)	−0.0062 (4)

### Geometric parameters (Å, °)

**Table:**

C1—C2	1.369 (9)	C11—H11	0.9500
C1—O1	1.378 (8)	C12—C13	1.483 (9)
C1—C10	1.422 (9)	C12—H12	0.9500
C2—C3	1.420 (9)	C13—O2	1.238 (8)
C2—H2	0.9500	C13—C14	1.489 (10)
C3—C4	1.407 (9)	C14—C19	1.389 (9)
C3—C8	1.436 (9)	C14—C15	1.406 (9)
C4—C5	1.364 (9)	C15—C16	1.361 (9)
C4—H4	0.9500	C15—H15	0.9500
C5—C6	1.439 (9)	C16—C17	1.391 (10)
C5—H5	0.9500	C16—Br1	1.909 (7)
C6—C7	1.387 (9)	C17—C18	1.379 (9)
C6—C11	1.459 (9)	C17—H17	0.9500
C7—C8	1.407 (9)	C18—C19	1.380 (10)
C7—H7	0.9500	C18—H18	0.9500
C8—C9	1.414 (9)	C19—H19	0.9500
C9—C10	1.366 (9)	C20—O1	1.418 (8)
C9—H9	0.9500	C20—H20A	0.9800
C10—H10	0.9500	C20—H20B	0.9800
C11—C12	1.333 (9)	C20—H20C	0.9800
			
C2—C1—O1	126.3 (6)	C6—C11—H11	116.4
C2—C1—C10	120.8 (6)	C11—C12—C13	122.0 (7)
O1—C1—C10	112.9 (6)	C11—C12—H12	119.0
C1—C2—C3	119.7 (6)	C13—C12—H12	119.0
C1—C2—H2	120.1	O2—C13—C12	120.3 (7)
C3—C2—H2	120.1	O2—C13—C14	121.1 (7)
C4—C3—C2	122.2 (6)	C12—C13—C14	118.6 (6)
C4—C3—C8	118.1 (6)	C19—C14—C15	119.2 (7)
C2—C3—C8	119.7 (6)	C19—C14—C13	122.2 (7)
C5—C4—C3	122.0 (6)	C15—C14—C13	118.5 (6)
C5—C4—H4	119.0	C16—C15—C14	120.2 (7)
C3—C4—H4	119.0	C16—C15—H15	119.9
C4—C5—C6	120.9 (6)	C14—C15—H15	119.9
C4—C5—H5	119.6	C15—C16—C17	121.2 (7)
C6—C5—H5	119.6	C15—C16—Br1	120.6 (5)
C7—C6—C5	117.6 (6)	C17—C16—Br1	118.2 (5)
C7—C6—C11	120.5 (6)	C18—C17—C16	118.1 (7)
C5—C6—C11	121.7 (6)	C18—C17—H17	120.9
C6—C7—C8	122.4 (6)	C16—C17—H17	120.9
C6—C7—H7	118.8	C17—C18—C19	122.1 (7)
C8—C7—H7	118.8	C17—C18—H18	119.0
C7—C8—C9	122.5 (7)	C19—C18—H18	119.0
C7—C8—C3	119.0 (6)	C18—C19—C14	119.1 (8)
C9—C8—C3	118.5 (6)	C18—C19—H19	120.4
C10—C9—C8	120.8 (7)	C14—C19—H19	120.4
C10—C9—H9	119.6	O1—C20—H20A	109.5
C8—C9—H9	119.6	O1—C20—H20B	109.5
C9—C10—C1	120.4 (7)	H20A—C20—H20B	109.5
C9—C10—H10	119.8	O1—C20—H20C	109.5
C1—C10—H10	119.8	H20A—C20—H20C	109.5
C12—C11—C6	127.2 (7)	H20B—C20—H20C	109.5
C12—C11—H11	116.4	C1—O1—C20	115.6 (6)
			
O1—C1—C2—C3	−180.0 (6)	C7—C6—C11—C12	−178.5 (7)
C10—C1—C2—C3	−1.4 (9)	C5—C6—C11—C12	7.2 (10)
C1—C2—C3—C4	−178.0 (6)	C6—C11—C12—C13	−177.3 (6)
C1—C2—C3—C8	2.7 (9)	C11—C12—C13—O2	23.0 (11)
C2—C3—C4—C5	−177.7 (6)	C11—C12—C13—C14	−154.4 (6)
C8—C3—C4—C5	1.6 (9)	O2—C13—C14—C19	−163.5 (7)
C3—C4—C5—C6	0.2 (10)	C12—C13—C14—C19	13.8 (10)
C4—C5—C6—C7	−1.1 (9)	O2—C13—C14—C15	12.6 (10)
C4—C5—C6—C11	173.4 (6)	C12—C13—C14—C15	−170.1 (6)
C5—C6—C7—C8	0.1 (9)	C19—C14—C15—C16	1.2 (10)
C11—C6—C7—C8	−174.5 (6)	C13—C14—C15—C16	−175.0 (6)
C6—C7—C8—C9	−179.1 (6)	C14—C15—C16—C17	−1.5 (10)
C6—C7—C8—C3	1.7 (10)	C14—C15—C16—Br1	176.6 (5)
C4—C3—C8—C7	−2.5 (9)	C15—C16—C17—C18	1.9 (10)
C2—C3—C8—C7	176.8 (6)	Br1—C16—C17—C18	−176.2 (5)
C4—C3—C8—C9	178.3 (6)	C16—C17—C18—C19	−2.2 (10)
C2—C3—C8—C9	−2.4 (9)	C17—C18—C19—C14	2.0 (10)
C7—C8—C9—C10	−178.3 (6)	C15—C14—C19—C18	−1.5 (10)
C3—C8—C9—C10	0.9 (10)	C13—C14—C19—C18	174.6 (6)
C8—C9—C10—C1	0.4 (10)	C2—C1—O1—C20	1.2 (9)
C2—C1—C10—C9	−0.2 (10)	C10—C1—O1—C20	−177.5 (6)
O1—C1—C10—C9	178.6 (6)		

### Hydrogen-bond geometry (Å, °)

**Table:**

Cg2 is the centroid of the C3–C8 ring.

**Table:**

*D*—H···*A*	*D*—H	H···*A*	*D*···*A*	*D*—H···*A*
C4—H4···Cg2^i^	0.95	2.70	3.432 (6)	134
C7—H7···Cg2^ii^	0.95	2.80	3.520 (6)	134

Symmetry codes: (i) −*x*+1/2, *y*−3/2, *z*; (ii) −*x*+1, *y*+1/2, −*z*+1/2.
